# Clinical performance of short fiber-reinforced and indirect resin composites in class I and class II restorations: A three-year randomized clinical trial

**DOI:** 10.1007/s00784-025-06593-x

**Published:** 2025-11-21

**Authors:** Rasha M. Salama, Hamdi H. Hamama, Salah H. Mahmoud

**Affiliations:** 1 Conservative Dentistry Department, Faculty of Dentistry, Mansoura University, Mansoura, Egypt; 2 Conservative Dentistry Department, Faculty of Dentistry, Kafr El-Sheikh University, Kafr El-Sheikh, Egypt; 3 Faculty of Oral and Dental Medicine, Alsalam University, Tanta, Egypt

**Keywords:** Indirect composite, Lab composite, Microhybrid composite, Short fiber-reinforced composite

## Abstract

**Objective:**

This prospective randomized clinical trial was designed to evaluate and compare the three-year clinical performance of short fiber-reinforced composite (SFRC) and indirect laboratory (lab) composite with that of a microhybrid resin composite placed in Class I and Class II cavities, with marginal adaptation defined as the primary outcome.

**Materials and methods:**

Thirty-three participants, each exhibiting 3 posterior carious teeth (Class I or II, ICDAS 4 or 5) under stable occlusion, were enrolled in this study. A total of 99 restorations (33 for each material) were placed as follows: SFRC (everX Posterior), indirect lab composite (SR Nexco), and microhybrid resin composite (G-aenial Posterior). Clinical assessments were conducted at baseline (1 week), 6 months, 1, 2, and 3 years by two blinded examiners utilizing FDI criteria, with marginal adaptation designated as the primary outcome, whereas other FDI criteria were assessed as secondary outcomes. Intragroup differences across follow-ups were assessed using Friedman and Wilcoxon post-hoc tests, while intergroup comparisons were analyzed using Kruskal-Wallis and Mann-Whitney U tests. The significance level was set at α = 0.05.

**Results:**

Twenty-eight patients with a total of 84 restorations were evaluated at the end of the 3-years with 84.45% recall rates. The outcomes revealed no statistically significant differences among SFRC, indirect lab composite, and microhybrid resin composite restorations for the assessed criteria (*p* > 0.05). The clinical success rates for SFRC (everX Posterior), indirect lab composite (SR Nexco), and microhybrid resin composite (G-aenial Posterior) were 100%, 100%, and 96.43%, respectively.

**Conclusion:**

After a three-year follow-up period, both SFRC and indirect lab composite demonstrated acceptable clinical performance, comparable to that of microhybrid resin composite, as evaluated by the FDI criteria.

**Clinical significance:**

Although the tested resin composite materials revealed similar clinical behavior in posterior teeth, the prolonged treatment time and higher cost of indirect lab composite favor the direct approach.

**Supplementary Information:**

The online version contains supplementary material available at 10.1007/s00784-025-06593-x.

## Introduction

Posterior restorations are crucial for preserving oral health, as they contribute to dental arch stability, maintain proper occlusal function, and support the structural integrity of the teeth. Nevertheless, teeth situated in the posterior region may endure high forces under certain conditions, such as bruxism or biting on hard objects [1–3]. Direct resin composite restorations, with their excellent esthetic properties, constitute the primary modality in restorative dentistry that has been successfully utilized for many years [4]. Despite this, insufficient material properties limited their success in high-stress bearing areas [5, 6]. They are associated with polymerization shrinkage, inadequate polymerization in deep interproximal areas, and difficult restoration of proximal contacts and contours [7–10]. These limitations contribute to clinical failures, with fractures being the predominant cause during the one to four-year follow-up periods, followed by secondary caries [11–13]. 

Accordingly, advanced research has been conducted to improve resin composite materials, aiming to achieve a material that possesses superior strength and low polymerization shrinkage. One of the advancements in resin composite technology is the evolution of short fiber-reinforced composite (SFRC), where the filler system is potentiated with short glass fibers, emulating the fibrous nature of dentin where collagen fibers are integrated inside the matrix [3, 14]. These fibers have the ability to absorb stresses and disperse energy, functioning as crack stoppers and preventing brittle fracture of the material [15, 16]. This leads to improved mechanical performance, offering a viable approach for reinforcing structurally compromised teeth in high-stress bearing areas and asserting to overcome the drawbacks of conventional composites [17]. Furthermore, SFRC mitigate marginal microleakage by modulating the stresses induced by polymerization shrinkage through fiber orientation. This stress reduction helps preserve the integrity of the tooth-restoration interface, thereby enhancing marginal adaptation and minimizing the risk of marginal deterioration over time [18]. Previous in vitro studies have demonstrated favorable outcomes associated with the incorporation of fiber reinforcement in resin composite restorations [1, 19–22]. 

Indirect composite restorative systems were also introduced as an alternative for clinicians to overcome some of the drawbacks associated with direct placement [7, 23, 24]. Their polymerization process is performed in a laboratory, resulting in an enhanced degree of conversion and reduced polymerization shrinkage, limiting it to the thin luting cement layer. They also allow increased wear resistance, greater control of form and function, especially in areas with limited accessibility, better occlusal contacts, and improved fracture resistance [25–27]. Nonetheless, prior clinical research has demonstrated that indirect composite restorations yielded conflicting and debatable results when compared to their direct counterparts [7, 8, 28–30]. Despite the growing popularity and acceptable performance of single-visit CAD/CAM restorations for moderate posterior lesions, indirect lab composites remain widely used in daily clinical practice, particularly in regions where digital workflows are not yet fully implemented or economically feasible [31]. 

Laboratory data establishes a fundamental basis for examining the effects of different restorative materials under controlled conditions; however, only clinical trials can thoroughly evaluate all the potential variables present in the oral cavity [32]. While SFRC represents an innovative approach, there remains a paucity of evidence assessing their clinical performance. This limited evidence highlights the need for further investigation to ascertain their long-term efficacy and durability compared to other materials. In the present study, the research question was structured based on the PICO model: P (Population): patients requiring Class I and Class II posterior composite restorations; I (Intervention): included SFRC and indirect lab composite restorations; C (Comparison): microhybrid resin composite restorations; and O (Outcome): marginal adaptation as the primary outcome, with other FDI criteria assessed as secondary outcomes. Accordingly, this randomized clinical trial was designed to evaluate and compare the three-year clinical performance of SFRC and indirect lab composite with that of a microhybrid resin composite placed in Class I and Class II cavities, with marginal adaptation defined as the primary outcome. The null hypothesis stated that the three restorative materials would exhibit comparable clinical performance according to FDI criteria.

## Materials & methods

### Study design

The ethics committee of Faculty of Dentistry, Mansoura University granted ethical approval (no. A02071221) to this study prior to commencement. A comprehensive elucidation of all the procedures entailed in this study was provided to the patients before obtaining their informed consent for participating, in accordance with the Helsinki protocols. The trial was registered in the clinical trial registration database (www.clinicaltrials.gov) under identification number (NCT06803537). The initial protocol version was registered and no post-registration modifications were made. The present study implemented a split-mouth prospective double-blinded (including both patients and examiners), randomized controlled clinical trial, which is in conformity with the consolidated standards of reporting trials (CONSORT) statement [33]. The initial interventions were performed in January 2022, and the final recall was completed in December 2024. The study was conducted as an integral component of a doctoral dissertation.

### Restorative materials and study groups

Three different restorative materials were utilized in this study: SFRC (everX Posterior, GC, Tokyo, Japan), indirect lab composite (SR Nexco, Ivoclar Vivadent AG, Schaan, Liechtenstein), and microhybrid resin composite (G-aenial Posterior, GC, Tokyo, Japan). The full description of the materials is presented in Table 1. They were equally allocated into three groups, with the first two restorative materials designated as the test groups and the microhybrid resin composite serving as the control group, as follows:

- Group 1: Direct restorations using SFRC (everX Posterior, GC, Tokyo, Japan), capped with a 1 mm occlusal layer of microhybrid resin composite (G-aenial Posterior, GC).
- Group 2: Indirect lab composite (SR Nexco, Ivoclar Vivadent AG), cemented with a universal dual-curing resin cement (Multilink N, Ivoclar Vivadent AG).
- Group 3: Direct restorations using microhybrid resin composite (G-aenial Posterior, GC, Tokyo, Japan), placed incrementally.

**Table 1:** Materials used in the study

Material	Composition	Manufacturer	Batch No.
EverX Posterior	Bis-GMA, PMMA, TEGDMA, short E-glass fiber filler, barium glass (76 wt%, 57 vol%)	GC, Tokyo, Japan	2,110,011
G-aenial Posterior	UDMA, dimethacrylate comonomers, silica& strontium fluoride containing fillers (80 wt%, 65 vol%)	GC, Tokyo, Japan	210,708 A
G-Premio BOND	MDP, MDTP, 4-MET, BHT, acetone, water, dimethacrylates monomers, photoinitiators, silica fillers, pH:1.5	GC, Tokyo, Japan	2,103,171
N-Etch	37% phosphoric acid gel	Ivoclar Vivadent AG, Schaan, Liechtenstein	Z04HGF
SR Nexco paste Layering materials (incisal & dentin)	Dimethacrylates (17–19 wt%), copolymer, silicon dioxide (82–83 wt%), Inoraganic filler (64–65 wt%)	Ivoclar Vivadent AG, Schaan, Liechtenstein	SR Nexco Incisal Z02Y74 SR Nexco Dentin Z02WHT
SR Nexco liner	Dimethacrylates (48 wt%), barium glass filler, silicon dioxide (51 wt%)	Ivoclar Vivadent AG, Schaan, Liechtenstein	Z02209
SR Gel	Glycerine, silicon dioxide, aluminium oxide	Ivoclar Vivadent AG, Schaan, Liechtenstein	Z03254
Universal Polishing Paste	Aluminium oxide, ammonium oleate, petroleum distillate, water	Ivoclar Vivadent AG, Schaan, Liechtenstein	XL4210
Multilink N	Dimethacrylates, HEMA, barium glass, ytterbium trifluoride, mixed oxide (vol 40%)	Ivoclar Vivadent AG, Schaan, Liechtenstein	Z040GN
Multilink N primer A	Aqueous solution of initiators	Ivoclar Vivadent AG, Schaan, Liechtenstein	Z051VR
Multilink N primer B	HEMA, phosphoric acid, methacrylate monomers	Ivoclar Vivadent AG, Schaan, Liechtenstein	Z055MM
Monobond N	Alcohol solution of silane methacrylate, phosphoric acid methacrylate, sulphide methacrylate	Ivoclar Vivadent AG, Schaan, Liechtenstein	Z04S5G

4-MET: 4-Methacryloxyethyl Trimellitate, BHT: Butylated HydroxyToluene, Bis-GMA: Bisphenol A-Glycidyl Methacrylate, HEMA: HydroxyEthyl MethAcrylate, MDP: Methacryloyloxydecyl Dihydrogen Phosphate, MDTP: Methacryloyloxydecyl Thiophosphoryl Methacrylate, PMMA: PolyMethyl MethAcrylate, TEGDMA: TriEthylene Glycol DiMethAcrylate, UDMA: Urethane DiMethAcrylate

### Sample size calculation

The statistical software G*Power was utilized for estimating the appropriate sample size. Based on a level of significance of 0.05, an effect size of 0.5, and a power of 80, it was determined that a sample size of 27 per group was required [34]. This calculation was based on the assumed effect size for the primary outcome, marginal adaptation. The overall sample size was expanded to 33 patients with a total of 99 restorations to account for any dropouts.

### Patient selection

The current study recruited thirty-three adult patients who were seeking dental treatment at the Faculty of Dentistry’s Outpatient Clinic, Mansoura University. One evaluator conducted the preliminary assessment to find out if the individuals comply with the inclusion demands. The medical and dental histories of the patients were documented, followed by a clinical examination. Vitality tests and radiographic examinations were performed when deemed necessary to exclude teeth that required endodontic treatment.

### Eligibility criteria

#### Inclusion criteria

The study included patients aged 18–35 years of both genders with good oral hygiene, categorized as low to moderate caries risk based on the CAMBRA (Caries Management by Caries Risk Assessment) protocol. Eligible participants presented at least three primary occlusal or proximal carious lesions (Black Class I or II) with an ICDAS (International Caries Detection and Assessment System) severity score of 4 or 5 upon visual examination. The carious teeth had to be vital, without periapical radiolucency (confirmed by periapical radiography), and in stable occlusion.

#### Exclusion criteria

Patients were excluded if they exhibited extremely poor oral hygiene, uncontrolled systemic diseases, chronic periodontitis, or heavy bruxism. Further exclusion criteria comprised extensive cavities exceeding two-thirds of the intercuspal width, lesions requiring cusp coverage, ongoing orthodontic treatment, or inability to attend scheduled follow-up appointments.

### Randomization & allocation concealment

Randomization was performed using an online software (https://www.randomizer.org). A randomization code was generated according to the three treatment possibilities. Each patient received three different posterior restorations in a unique sequence determined by the developed random sequence plan. Each restoration was planned to be placed in a separate quadrant to maintain the diagnostic clarity provided by the split-mouth design. In the few instances where clinical factors (e.g. the distribution of carious lesions) necessitated placing two restorations in the same quadrant, they were positioned non-adjacently and separated by at least one unrestored tooth to preserve the ability to localize symptoms [35]. To ensure allocation concealment, the randomization sequence was secured using sequentially numbered, opaque, sealed envelopes, which were prepared by an independent coordinator not involved in the clinical procedures or outcome evaluation. Each envelope was opened only after confirming patient eligibility and obtaining informed consent. Blinding was implemented for both the patients and the outcome assessors; however, blinding the operator was not feasible due to the inherent differences in material composition and the distinct application techniques required for each restorative system.

### Clinical procedures

The operative procedures were conducted by a sole operator who possessed 7 years of training and expertise. Preoperative digital photographs were taken as part of the dental screening. Patients were administered local anesthesia prior to restorative procedures in order to alleviate pain and discomfort. Fluoride-free prophylaxis paste was used for cleansing the teeth of all participants. Subsequently, the operative field was isolated using rubber dam and high suctioning.

#### Direct restorations

The initial cavity preparation was performed using suitably sized carbide straight fissure burs (Komet, Lemgo, Germany) in a high-speed handpiece (Sirona T3, Bensheim, Germany) while maintaining a constant, copious air-water cooling system. The extent of caries dictated the preparation design, without beveling the walls or margins, and without involving any cusps. The remaining carious infected dentin, if present, was removed using a sharp manual excavator (#52; Dentsply Maillefer, Switzerland). Selective caries removal was performed by carefully removing only infected dentin while preserving affected dentin in deep cavities, in accordance with minimally invasive principles. The cavity preparation was completed using a finishing diamond, with the cavity walls prepared conservatively according to lesion extent and slightly rounded line angles. In deep cavities where the remaining dentin thickness was estimated to be < 1 mm, pulp protection was provided using a thin layer of calcium hydroxide liner (Dycal, Dentsply, USA). The liner was applied in a thin layer over the deepest part of the cavity floor and light-cured for 20 s before adhesive application.

Selective enamel etching was performed by applying 37% phosphoric acid gel (N-Etch, Ivoclar Vivadent AG) to the enamel margins for 20 s prior to adhesive application, while dentin was left unetched, following the manufacturer’s instructions. Each cavity was then thoroughly rinsed with water for 20 s and gently air-dried, retaining the dentin surface with a slightly moist appearance. G-Premio BOND universal adhesive (GC, Tokyo, Japan) was applied to the prepared enamel and dentin surfaces and left undisturbed for 10 s. A gentle stream of air was then applied for 5 s to ensure thorough evaporation of the solvent and create a uniform adhesive film. Light curing was performed for 20 s using a light-emitting diode (LED) curing unit (Ledition, Ivoclar Vivadent AG), with a wave length between 430 and 490 nm. The light intensity of the unit was monitored with a dental radiometer (Bluephase meter, Ivoclar Vivadent AG) at 600 mW/cm^2^. In Class II preparations, the proximal wall was restored utilizing a horizontal incremental technique. A pre-contoured sectional matrix system (TOR VM, Russia), secured with an appropriate ring and properly sized wedge, was used to achieve optimal anatomical contact and contour. The missing wall was reestablished by placing a thin horizontal increment of microhybrid resin composite (G-aenial Posterior, GC, Tokyo, Japan) against the matrix, followed by light-curing for 40 s.

**Fig. 1:**
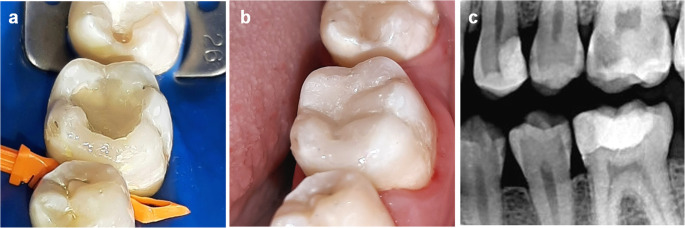
Restoration of tooth #36: **(a)** Placement of SFRC base (everX Posterior), **(b)** Final restoration after layering microhybrid resin composite (G-aenial Posterior), and **(c)** Postoperative bitewing radiograph

Following the manufacturer’s recommendations, SFRC (everX Posterior, GC, Tokyo, Japan) was applied in one increment, leaving 1 mm space for a surface layer of microhybrid resin composite, followed by 40 s of light polymerization for each (Fig. 1). However, in the microhybrid resin composite group (G-aenial Posterior, GC, Tokyo, Japan), the composite was applied incrementally, with each increment light-cured for 40 s from the occlusal aspect. Following the removal of the matrices, all restorations were additionally light-polymerized to ensure adequate curing of the proximal margins. After removal of the rubber dam, static and dynamic occlusion were assessed utilizing articulating paper, then finishing was done using fine grit diamond stones to remove any gross overhangs. Polishing procedures were performed implementing a low-speed handpiece with silicon carbide impregnated cups and points (KENDA AG, Vaduz, Liechtenstein) under continuous water cooling, following the manufacturer’s recommended sequential protocol. For Class II cavities, interdental flossing was employed to assess the tightness of proximal contacts and to ensure the absence of flashes or overhangs.

#### Indirect restorations

 Inlay cavity preparations were performed utilizing a specialized inlay preparation kit (Komet, Brasseler GmbH & Co. KG, Lemgo, Germany) in order to attain an estimated 10^◦^−12^◦^ occlusal divergence angles. A preliminary impression was obtained for each patient utilizing equal proportions of the base and catalyst of high-viscosity impression material (Presigum Putty, PD GmbH, Allershausen, Germany). Afterwards, a final impression was taken for each cavity using light-viscosity impression paste (Presigum Low viscosity, PD GmbH, Allershausen, Germany). Provisional restorations were applied using light-curing, eugenol-free temporary restorative material (Systemp inlay; Ivoclar Vivadent AG, Schaan, Liechtenstein). Each impression was then delivered to the dental laboratory for casting into a die stone.

##### Inlays fabrication

A professional dental technician fabricated all the restorations on the die stone, following the manufacturer’s guidelines. The die margins were precisely delineated using a red pencil, and then any undercuts were blocked with wax to guarantee easy removal of the restoration after the polymerization process without damaging the die model. The model sealer was applied in order to harden the stone surface and protect the die model. SR model Separator (Ivoclar Vivadent AG, Schaan, Liechtenstein) was placed in two thin coats. SR Nexco Liner was applied in a thick layer on the cavity walls and floor, and each segment was light-cured for 20 s. The cavity restoration began with incremental placement of SR Nexco Dentin, where the first increment was adapted firmly to ensure an effective bond between the liner and the laboratory composite. Each segment was light polymerized for 20 s, then SR Nexco Incisal was applied and also cured for 20 s. Following the layering procedure, all restorations were cured for 20 s in each direction. SR Nexco Gel was applied on the outer surface of the restorations, ensuring that all areas were completely coated, and then the restorations were placed in the furnace (Targis Power TP3 Upgrade, Ivoclar Vivadent AG) for final polymerization using program P2 for 11 min, as recommended by the manufacturer. Each inlay was carefully removed from the die model before being finished with fine diamonds and carbide burs under low speed and light pressure. The restorations were then polished with leather buffing wheels and Universal Polishing Paste (Ivoclar Vivadent AG).

##### Inlays cementation

Inlays cementation was conducted under rubber dam isolation and high suctioning. In order to achieve an excellent bond with the luting composite, the internal surfaces of the inlays were carefully sandblasted with 80–100 μm Al_2_O_3_ at 1 bar pressure, then they were conditioned by applying a thin layer of universal priming agent (Monobond N) and allowed to react for 60 s. The two primer liquids, Multilink N Primer A and B, were mixed together in equal parts on a mixing pad, then applied to the entire cavity with 30 s scrubbing, and the excess was dispersed with air until the mobile liquid film was no longer visible. Multilink N cement was applied directly to the inner surface of the restoration, then the restoration was seated rapidly in place. The excess material was removed using a foam pellet, followed by additional light curing to all margins for 20 s. Subsequently, the occlusion was assessed using articulating papers and the restorations were finished with flexible discs (Sof-Lex XT Pop On, 3 M ESPE) using the recommended sequence.

### Clinical evaluation

 Two blinded assessors evaluated the restorations clinically utilizing the FDI criteria. Prior to this, they went through training and calibration by analyzing clinical cases that had comparable features. For the results of a study to be valuable, it is imperative that the examiners reach a consensus over its interpretation. Therefore, both inter- and intra-examiner agreement were measured using Cohen’s Kappa coefficient, with 0.85 as the minimum value to be requested for reliability.

The participants were recalled for baseline evaluation after a week, followed by further assessments at 6 months, 1-year, 2-years, and 3-years. All participants adhered to the assigned treatment protocols, and no major protocol deviations occurred during the study period. The assessed criteria included functional properties such as marginal adaptation, material fracture, and the quality of proximal contact and contour. Additionally, biological properties including postoperative hypersensitivity, caries around restoration margins, and tooth integrity were also considered. Finally, esthetic properties as surface luster and texture, marginal staining, and color matching were evaluated. Among these, marginal adaptation was designated as the primary outcome, while all other assessed FDI criteria were considered secondary outcomes.

The restorations were categorized utilizing the following ranking terms: clinically excellent or very good, clinically good, clinically satisfactory, clinically unsatisfactory, and clinically poor. Rankings of 1, 2, and 3 were designated as “clinically successful,” while 4 and 5 were seen as indicative of failure. The parameters that required visual examination were conducted using a magnifying dental loupe, with a powerful attached light source. Marginal adaptation was assessed using two specialized blunt-tip probes (150 μm and 250 μm) in conjunction with dental floss for comprehensive evaluation. Postoperative sensitivity was evaluated by blowing a stream of cold air for 3 s at a distance of 2–3 cm from the restoration. Clinical intraoral photographs were taken at each follow-up appointment to monitor any visual alterations in the restorations.

### Statistical analysis

 The collected data were tabulated, coded, and statistically analyzed using IBM SPSS Statistics software (Version 29.0; IBM Corp., Armonk, NY, USA). Pearson’s Chi-Square was utilized to assess the distribution of data and the homogeneity in the clinical features of the restored cavities. The Friedman test was conducted to evaluate intragroup comparisons of the same restoration outcomes through different follow-up periods. Afterwards, the Wilcoxon signed-rank test was applied to ascertain the groups accountable for any significant variance. The Kruskal-Wallis test was utilized to assess intergroup comparisons between different restorations during the same follow-up period, followed by multiple comparisons employing the Mann-Whitney U test. A per-protocol analysis approach was applied, where only restorations with complete three-year follow-up data were included in the final statistical evaluation, and drop-outs were excluded from analysis. A significance level of 5% was used to analyze the comparisons.

## Results

**Fig. 2:**
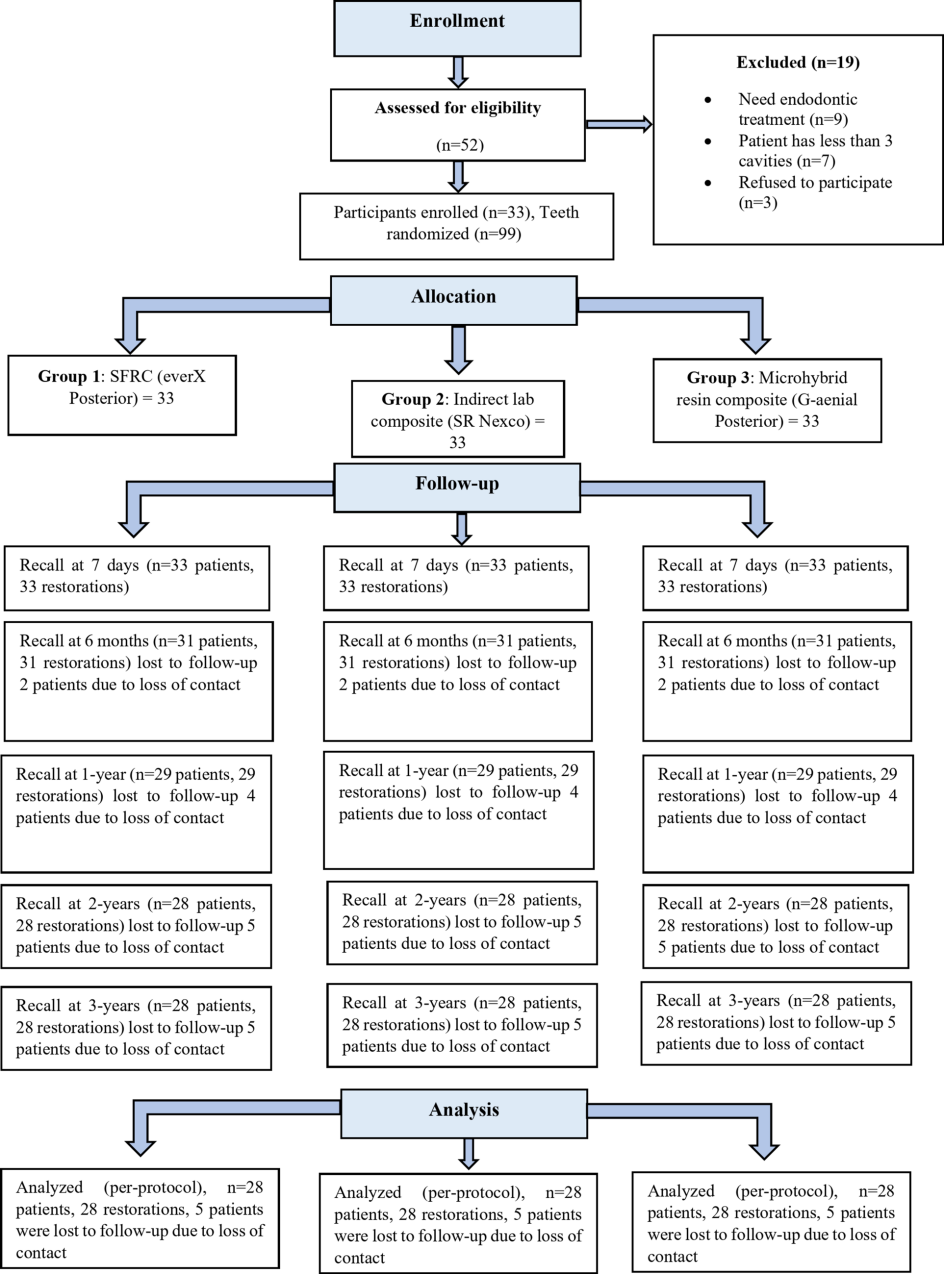
CONSORT flow diagram

 In this study, 52 participants were assessed for eligibility, with 19 of them being eliminated from the study because they either required endodontic treatment, encountered less than 3 posterior cavities, or declined to participate. Only those participants who reached a complete consensus were included in the trial. The representation of the recruiting, allocation, and follow-up periods for the patients is illustrated in Fig. 2.

### Recall rates

 Thirty-three patients, including 23 females and 10 males, were enrolled in this study. The mean age of the patients was 25.7 years. The study’s recall rates were as follows: 100% at baseline, 93.94% at six months, 87.88% at one year, and 84.85% at both two and three years. Five patients were lost to follow-up due to loss of contact despite multiple recall attempts through phone calls and text messaging.

### Evaluators agreement

 The Cohen’s Kappa statistics showed strong inter-examiner agreement at baseline assessment (0.95), as well as after 6 months (0.94), 1 year (0.92), 2 years (0.91), and 3 years (0.90). Strong intra-examiner agreement (ranging from 0.89 to 0.94) was also demonstrated throughout the evaluation periods.

### Characteristics of the restored cavities

The clinical parameters distribution of the restored cavities and the results of Pearson’s Chi-square test are illustrated in Table 2. All parameters (Black classification, teeth distribution, dental arch distribution, pulp protection, width, depth, and reason for restoration) were uniformly distributed among the restorative materials (*p* > 0.05).

**Table 2:** Pearson’s Chi-square test results for the characteristics of restored cavities

Characteristics of restored teeth		Number of lesions			*p*-value
		SFRC (everX Posterior)	Indirect lab composite (SR Nexco)	Microhybrid resin composite (G-aenial Posterior)	
Black classification	Class I	9	7	11	0.519
	Class II	19	21	17	
Teeth distribution	Premolars	8	4	6	0.427
	Molars	20	24	22	
Dental arch distribution	Upper	9	8	5	0.449
	Lower	19	20	23	
Pulp protection	Yes	3	0	2	0.225
	No	25	28	26	
Width	Small (1/4 to < 1/3 intercuspal distance)	4	2	6	0.675
	Medium (1/3 to < 2/3 intercuspal distance)	24	26	22	
	Large (approximately 2/3 intercuspal distance)	0	0	0	
Depth	Shallow (RDT > 2 mm)	0	0	3	0.360
	Medium (RDT > 1–2 mm)	25	28	23	
	Deep (RDT < 1 mm)	3	0	2	
Reason for restoration	Fracture	0	0	0	0.982
	Caries	25	26	27	
	Caries and fracture	3	2	1	
	Esthetic	0	0	0	

### Clinical success rates

 After a three-year follow-up, both SFRC (everX Posterior) and indirect lab composite (SR Nexco) had a 100% success rate, whereas microhybrid resin composite (G-aenial Posterior) attained a success rate of 96.43%.

### Clinical performance according to the FDI criteria

#### Functional properties

**Fig. 3:**
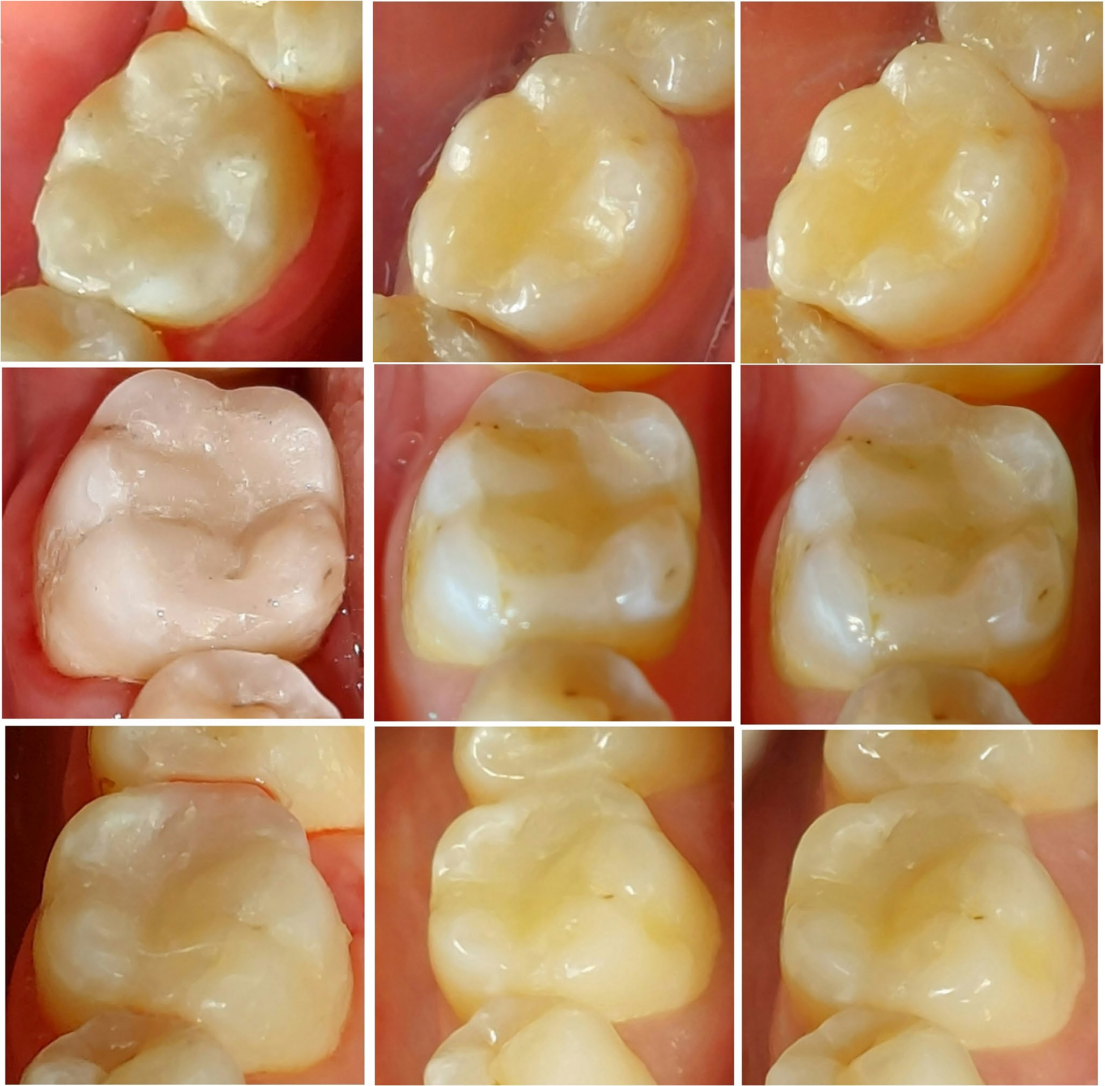
Representative clinical follow-up images illustrating marginal adaptation scores across the evaluation periods for the three restorative materials utilized in the study: **(a–c)** Tooth #46 restored with SFRC (everX Posterior) demonstrating FDI score 1 for marginal adaptation at baseline, 2-year, and 3-year evaluations respectively, **(d–f)** Tooth #46 restored with indirect lab composite (SR Nexco) exhibiting score 1 at baseline and score 2 at both 1-year and 2-year follow-ups, and **(g–i)** Tooth #36 restored with microhybrid resin composite (G-aenial Posterior) showing score 1 at baseline and 1-year, and score 2 for marginal adaptation at the 3-year follow-up

 Following a three-year follow-up period, the outcomes revealed no statistically significant differences among the three assessed restorative materials in terms of functional properties (*p* > 0.05). Concerning marginal adaptation, 92.9% of SFRC, 78.6% of indirect lab composite, and 85.7% of microhybrid resin composite restorations exhibited excellent marginal adaptation, scoring (1) Nevertheless, during the three-year follow-up assessment, one indirect lab composite restoration demonstrated marginal gaps and small fractures, which were assigned a score of 3. A statistically significant difference was observed for indirect lab composite restorations between the baseline and 1-year measurements, compared to 2-year and 3-year scores. For microhybrid resin composite restorations, the significant differences were observed between baseline and 3-year measurements (*p* < 0.05). Nonetheless, no significant difference was noted between the follow-up periods for SFRC (*p* > 0.05). Representative clinical images illustrating changes in marginal adaptation scores across the evaluation periods for the three restorative materials are presented in Fig. 3.

Regarding material fracture and retention, at the 3-year evaluation, SFRC restorations revealed ideal performance (100% scoring 1), whereas only one indirect lab composite restoration presented with hairline crack (score 2). At the 6-month follow-up, one microhybrid resin composite restoration exhibited minor fractures that were deemed clinically acceptable without compromising functionality, followed by another one at the two-year recall. However, by the end of the three-year duration, one microhybrid resin composite restoration was clinically unsatisfactory and required repair (score 4). The intragroup comparisons revealed no statistically significant differences between the follow-up periods for the three restorative materials (*p* > 0.05). All the restorations exhibited normal proximal contact and contour with no statistically significant differences detected at any follow-up evaluation in both intergroup and intragroup comparisons (*p* > 0.05). The results of the clinical functional assessments are presented in Table 3.

**Table 3:** Results of Kruskal-Wallis, Friedman and post hoc tests for functional characteristics according to FDI criteria

Category	FDI criteria	Time point		FDI score															*p*-value
				SFRC (everX Posterior)					Indirect lab composite (SR Nexco)					Microhybrid resin composite (G-aenial Posterior)					
				1	2	3	4	5	1	2	3	4	5	1	2	3	4	5	
(A) Functional Properties	Marginal adaptation	Baseline	n	28	0	0	0	0	28^ab^	0	0	0	0	28^a^	0	0	0	0	1.0
			%	100%	0	0	0	0	100%	0	0	0	0	100%	0	0	0	0	
		6-months	n	28	0	0	0	0	28^cd^	0	0	0	0	28	0	0	0	0	1.0
			%	100%	0	0	0	0	100%	0	0	0	0	100%	0	0	0	0	
		1-year	n	28	0	0	0	0	26	2	0	0	0	27	1	0	0	0	0.355
			%	100%	0	0	0	0	92.9%	7.1%	0	0	0	96.4%	3.6%	0	0	0	
		2-year	n	26	2	0	0	0	23^ac^	5	0	0	0	25	3	0	0	0	0.452
			%	92.9%	7.1%	0	0	0	82.1%	17.9%	0	0	0	89.3%	10.7%	0	0	0	
		3-year	n	26	2	0	0	0	22^bd^	5	1	0	0	24^a^	4	0	0	0	0.311
			%	92.9%	7.1%	0	0	0	78.6%	17.8%	3.6%	0	0	85.7%	14.3%	0	0	0	
		*p*-value		*p* = 0.092					*p* = 0.001*					*p* = 0.017*					
	Material fracture & retention	Baseline	n	28	0	0	0	0	28	0	0	0	0	28	0	0	0	0	1.0
			%	100%	0	0	0	0	100%	0	0	0	0	100%	0	0	0	0	
		6-months	n	28	0	0	0	0	28	0	0	0	0	27	1	0	0	0	0.363
			%	100%	0	0	0	0	100%	0	0	0	0	96.4%	3.6%	0	0	0	
		1-year	n	28	0	0	0	0	28	0	0	0	0	27	1	0	0	0	0.363
			%	100%	0	0	0	0	100%	0	0	0	0	96.4%	3.6%	0	0	0	
		2-year	n	28	0	0	0	0	28	0	0	0	0	26	2	0	0	0	0.129
			%	100%	0	0	0	0	100%	0	0	0	0	92.9%	7.1%	0	0	0	
		3-year	n	28	0	0	0	0	27	1	0	0	0	25	2	0	1	0	0.383
			%	100%	0	0	0	0	96.4%	3.6%	0	0	0	89.3%	7.1%	0	3.6%	0	
		*p*-value		*p* = 1.0					*p* = 0.406					*p* = 0.053					
	Proximal contact & contour	Baseline	n	19	0	0	0	0	21	0	0	0	0	17	0	0	0	0	1.0
			%	100%	0	0	0	0	100%	0	0	0	0	100%	0	0	0	0	
		6-months	n	19	0	0	0	0	21	0	0	0	0	17	0	0	0	0	1.0
			%	100%	0	0	0	0	100%	0	0	0	0	100%	0	0	0	0	
		1-year	n	19	0	0	0	0	21	0	0	0	0	17	0	0	0	0	1.0
			%	100%	0	0	0	0	100%	0	0	0	0	100%	0	0	0	0	
		2-year	n	19	0	0	0	0	21	0	0	0	0	17	0	0	0	0	1.0
			%	100%	0	0	0	0	100%	0	0	0	0	100%	0	0	0	0	
		3-year	n	19	0	0	0	0	21	0	0	0	0	17	0	0	0	0	1.0
			%	100%	0	0	0	0	100%	0	0	0	0	100%	0	0	0	0	
		*p*-value		*p* = 1.0					*p* = 1.0					*p* = 1.0					

Similar superscripted letters in the same column denote significant difference between different follow-up periods

#### Biological properties

No statistically significant differences were detected in biological characteristics among the three restorative materials after three years (*p* > 0.05). Similarly, intragroup comparisons demonstrated no significant differences between the follow-up durations among the assessed restorative materials (*p* > 0.05). Regarding postoperative hypersensitivity, indirect lab composite restorations revealed no sensitivity throughout the evaluation periods. However, at the 6-month evaluation, two SFRC restorations exhibited minor sensitivity, which was temporary and subsided shortly afterward. Furthermore, one microhybrid resin composite restoration revealed mild transient hypersensitivity at the 1-year follow-up, followed by two restorations at the 3-year follow-up. Concerning secondary caries and tooth integrity, no statistically significant differences were observed between the three restorative materials at any follow-up evaluation in both intragroup and intergroup comparisons (*p* > 0.05). The findings of the clinical biological evaluation are demonstrated in Table 4.

**Table 4:** Results of Kruskal-Wallis, Friedman and post hoc tests for biological characteristics according to FDI criteria

Category	FDI criteria	Time point		FDI score															*p*-value
				SFRC (everX Posterior)					Indirect lab composite (SR Nexco)					Microhybrid resin composite (G-aenial Posterior)					
				1	2	3	4	5	1	2	3	4	5	1	2	3	4	5	
(B) Biological properties	Post-operative hyper-sensitivity and tooth vitality	Baseline	n	28	0	0	0	0	28	0	0	0	0	28	0	0	0	0	1.0
			%	100%	0	0	0	0	100%	0	0	0	0	100%	0	0	0	0	
		6-months	n	26	2	0	0	0	28	0	0	0	0	28	0	0	0	0	0.129
			%	92.9%	7.1%	0	0	0	100%	0	0	0	0	100%	0	0	0	0	
		1-year	n	28	0	0	0	0	28	0	0	0	0	27	1	0	0	0	0.363
			%	100%	0	0	0	0	100%	0	0	0	0	96.4%	3.6%	0	0	0	
		2-year	n	28	0	0	0	0	28	0	0	0	0	28	0	0	0	0	1.0
			%	100%	0	0	0	0	100%	0	0	0	0	100%	0	0	0	0	
		3-year	n	28	0	0	0	0	28	0	0	0	0	26	2	0	0	0	0.129
			%	100%	0	0	0	0	100%	0	0	0	0	92.9%	7.1%	0	0	0	
		*p*-value		*p* = 0.092					*p* = 1.0					*p* = 0.171					
	Secondary caries	Baseline	n	28	0	0	0	0	28	0	0	0	0	28	0	0	0	0	1.0
			%	100%	0	0	0	0	100%	0	0	0	0	100%	0	0	0	0	
		6-months	n	28	0	0	0	0	28	0	0	0	0	28	0	0	0	0	1.0
			%	100%	0	0	0	0	100%	0	0	0	0	100%	0	0	0	0	
		1-year	n	28	0	0	0	0	28	0	0	0	0	28	0	0	0	0	1.0
			%	100%	0	0	0	0	100%	0	0	0	0	100%	0	0	0	0	
		2-year	n	28	0	0	0	0	28	0	0	0	0	28	0	0	0	0	1.0
			%	100%	0	0	0	0	100%	0	0	0	0	100%	0	0	0	0	
		3-year	n	28	0	0	0	0	28	0	0	0	0	28	0	0	0	0	1.0
			%	100%	0	0	0	0	100%	0	0	0	0	100%	0	0	0	0	
		*p*-value		*p* = 1.0					*p* = 1.0					*p* = 1.0					
	Tooth integrity (enamel cracks)	Baseline	n	28	0	0	0	0	28	0	0	0	0	28	0	0	0	0	1.0
			%	100%	0	0	0	0	100%	0	0	0	0	100%	0	0	0	0	
		6-months	n	28	0	0	0	0	28	0	0	0	0	28	0	0	0	0	1.0
			%	100%	0	0	0	0	100%	0	0	0	0	100%	0	0	0	0	
		1-year	n	28	0	0	0	0	28	0	0	0	0	28	0	0	0	0	1.0
			%	100%	0	0	0	0	100%	0	0	0	0	100%	0	0	0	0	
		2-year	n	28	0	0	0	0	28	0	0	0	0	28	0	0	0	0	1.0
			%	100%	0	0	0	0	100%	0	0	0	0	100%	0	0	0	0	
		3-year	n	28	0	0	0	0	28	0	0	0	0	28	0	0	0	0	1.0
			%	100%	0	0	0	0	100%	0	0	0	0	100%	0	0	0	0	
		*p*-value		*p* = 1.0					*p* = 1.0					*p* = 1.0					

#### Esthetic properties

The three restorative materials exhibited no statistically significant differences in terms of esthetic properties after three years (*p* > 0.05). Concerning surface luster and texture, minor surface changes were observed in two SFRC and three microhybrid resin composite restorations (score 2). No statistically significant differences were found between the follow-up durations among the three restorative materials, as demonstrated by intragroup comparisons (*p* > 0.05). Regarding marginal staining, it was observed in 14.3% of SFRC restorations, 21.4% of indirect lab composite restorations, and 25% of microhybrid resin composite restorations. These instances of staining were deemed clinically acceptable, receiving a score of (2) The three restorative materials demonstrated significant differences across the follow-up periods in intragroup comparisons (*p* < 0.05). After three years, 100% of indirect lab composite restorations, 92.9% of SFRC restorations, and 89.3% of microhybrid resin composite restorations were ideal in terms of color match. The outcomes of the clinical esthetic investigations are reported in Table 5.

**Table 5:** Results of Kruskal-Wallis, Friedman and post hoc tests for esthetic characteristics according to FDI criteria

Category	FDI criteria	Time point		FDI score															*p*-value
				SFRC (everX Posterior)					Indirect lab composite (SR Nexco)					Microhybrid resin composite (G-aenial Posterior)					
				1	2	3	4	5	1	2	3	4	5	1	2	3	4	5	
(C) Esthetic Properties	Surface luster and surface texture	Baseline	n	28	0	0	0	0	28	0	0	0	0	28	0	0	0	0	1.0
			%	100%	0	0	0	0	100%	0	0	0	0	100%	0	0	0	0	
		6-months	n	28	0	0	0	0	28	0	0	0	0	28	0	0	0	0	1.0
			%	100%	0	0	0	0	100%	0	0	0	0	100%	0	0	0	0	
		1-year	n	28	0	0	0	0	28	0	0	0	0	27	1	0	0	0	0.363
			%	100%	0	0	0	0	100%	0	0	0	0	96.4%	3.6%	0	0	0	
		2-year	n	27	1	0	0	0	28	0	0	0	0	27	1	0	0	0	0.599
			%	96.4%	3.6%	0	0	0	100%	0	0	0	0	96.4%	3.6%	0	0	0	
		3-year	n	26	2	0	0	0	28	0	0	0	0	25	3	0	0	0	0.226
			%	92.9%	7.1%	0	0	0	100%	0	0	0	0	89.3%	10.7%	0	0	0	
		*p*-value		*p* = 0.171					*p* = 1.0					*p* = 0.073					
	Marginal staining	Baseline	n	28^a^	0	0	0	0	28^a^	0	0	0	0	28^a^	0	0	0	0	1.0
			%	100%	0	0	0	0	100%	0	0	0	0	100%	0	0	0	0	
		6-months	n	27	1	0	0	0	28^b^	0	0	0	0	26^b^	2	0	0	0	0.355
			%	96.4%	3.6%	0	0	0	100%	0	0	0	0	92.9%	7.1%	0	0	0	
		1-year	n	25	3	0	0	0	27^c^	1	0	0	0	25	3	0	0	0	0.536
			%	89.3%	10.7%	0	0	0	96.4%	3.6%	0	0	0	89.3%	10.7%	0	0	0	
		2-year	n	25	3	0	0	0	25	3	0	0	0	24	4	0	0	0	0.893
			%	89.3%	10.7%	0	0	0	89.3%	10.7%	0	0	0	85.7%	14.3%	0	0	0	
		3-year	n	24^a^	4	0	0	0	22^abc^	6	0	0	0	21^ab^	7	0	0	0	0.597
			%	85.7%	14.3%	0	0	0	78.6%	21.4%	0	0	0	75%	25%	0	0	0	
		*p*-value		*p* = 0.029*					*p* = 0.002*					*p* = 0.002*					
	Color match	Baseline	n	28	0	0	0	0	28	0	0	0	0	28	0	0	0	0	1.0
			%	100%	0	0	0	0	100%	0	0	0	0	100%	0	0	0	0	
		6-months	n	28	0	0	0	0	28	0	0	0	0	28	0	0	0	0	1.0
			%	100%	0	0	0	0	100%	0	0	0	0	100%	0	0	0	0	
		1-year	n	28	0	0	0	0	28	0	0	0	0	26	2	0	0	0	0.129
			%	100%	0	0	0	0	100%	0	0	0	0	92.9%	7.1%	0	0	0	
		2-year	n	27	1	0	0	0	28	0	0	0	0	26	2	0	0	0	0.355
			%	96.4%	3.6%	0	0	0	100%	0	0	0	0	92.9%	7.1%	0	0	0	
		3-year	n	26	2	0	0	0	28	0	0	0	0	25	3	0	0	0	0.226
			%	92.9%	7.1%	0	0	0	100%	0	0	0	0	89.3%	10.7%	0	0	0	
		*p*-value		*p* = 0.171					*p* = 1.0					*p* = 0.061					

## Discussion

Randomized controlled trials (RCTs) are considered the gold standard for assessing healthcare interventions, as their explanatory capacity facilitates qualitative determinations on the impact of a treatment and a quantitative evaluation of its efficacy [36]. Although in vitro studies have revealed favorable mechanical outcomes regarding short fiber-reinforced composite (SFRC), comprehensive clinical trials concerning their long-term efficacy remain limited in the literature [1, 19–22]. Furthermore, previous studies have been constrained by their reliance on USPHS evaluation criteria, which may lack the discriminatory capacity to identify early material deterioration compared to contemporary FDI standards [16, 34, 37, 38]. This knowledge gap is compounded by the paucity of clinical studies directly comparing the performance of SFRC with both direct and indirect restorative approaches.

Consequently, this RCT was designed to expand the existing evidence base by evaluating SFRC for its promising marginal adaptation and enhanced mechanical properties in stress-bearing areas, while also assessing indirect lab composite as a viable solution to polymerization shrinkage, a persistent limitation of direct restorative materials [1, 3, 18, 39, 40]. The primary outcome of this clinical trial, marginal adaptation, was selected based on its crucial role in determining the longevity of posterior restorations. It reflects the long-term integrity of the tooth-restoration interface and serves as a reliable determinant of clinical success. Other assessed parameters, such as material fracture, which reflects the mechanical performance of the restorative material under masticatory stresses, were considered secondary outcomes that provide complementary insights into the overall clinical performance. To minimize inter-individual variability, a split-mouth design was employed under identical oral conditions [35]. Patients with high caries risk were precluded from this study, based on existing literature reporting reduced longevity and higher failure rates of composite restorations compared to those with low/moderate caries risk [41, 42]. Radiographs were not mandated as part of the follow-up protocol and were obtained only when clinically indicated, such as in the presence of symptoms or suspected pathology, to comply with the ALARA (As Low As Reasonably Achievable) principle, as the FDI criteria rely on direct intraoral visual and tactile assessment, rendering additional radiographic evaluation unnecessary in asymptomatic cases [43, 44]. The null hypothesis was accepted, as the SFRC, indirect lab composite and microhybrid resin composite restorations revealed comparable clinical performance in posterior dentition based on FDI evaluation criteria.

In the current study, SFRC restorations revealed favorable marginal adaptation, with 92.9% maintaining a score of 1; however, no notable differences were observed compared to the other evaluated materials. This aligns with prior research indicating that SFRC restorations had clinically acceptable marginal integrity. [16, 38, 45] Considering that polymerization stresses and insufficient depth of cure are the primary contributors to inadequate marginal adaptation, the aforementioned findings may be ascribed to the inclusion of glass fibers in SFRC composition, which facilitate light transmission and diffusion, allowing for adequate polymerization depth [46, 47]. The monomer composition of SFRC also includes a specialized group, N,N-dimethylaminoethyl methacrylate, which promotes polymerization by interacting with camphorquinone, with both compounds serving as photoinitiators [47]. Additionally, optimizing the aspect ratio, quantity, and random orientation of fibers in SFRC material can reduce shrinkage stress generation, as the intertwined fibers absorb the stresses induced by the contraction of the surrounding matrix, thereby enhancing stress-relieving capacity and potentially reducing stress levels at the tooth-restoration interface, thus improving marginal adaptation [48, 49]. On the contrary, a previous 3-year clinical trial reported that 12.5% of SFRC restorations failed due to fractures compromising marginal integrity, potentially attributable to patient- or operator-related factors [34].

On the other hand, indirect lab composite restorations exhibited the least favorable marginal adaptation among other restorative materials (21.4% scoring 2), showing a noticeable decline in marginal integrity over time but no notable differences from other groups. This observation aligns with a previous one-year clinical trial, which reported that indirect lab composite restorations demonstrated inferior marginal adaptation compared to SFRC restorations [16]. These outcomes may be ascribed to the nature of indirect restorations, which entail several restorative procedures, each representing a possible failure point, especially along the cement line, resulting in marginal openings. Nevertheless, direct restorations include fewer stages, enabling enhanced control over marginal adaptability [30, 50]. Also, the extraoral polymerization of indirect lab composite results in a high conversion rate and a crosslinked structure, leaving fewer double bonds available to react with the luting composite. This consequently impairs the bonding strength between indirect lab composite inlays and luting composite, thereby adversely impacting marginal adaptation [40, 51]. A further contributing factor in the present study may be the absence of immediate dentin sealing (IDS) prior to impression-taking for the indirect restorations. This decision was based on the aim to mimic the standard clinical routine followed by many practitioners and to maintain consistency with the manufacturer’s instructions. However, the lack of IDS may have contributed to the relatively lower marginal adaptation scores observed in the indirect group at later follow-ups. IDS has been reported in the literature to enhance the bond strength between dentin and resin cements, reduce postoperative sensitivity, and improve marginal sealing by protecting freshly cut dentin from bacterial contamination and dehydration before luting procedures [52]. Although indirect lab composite restorations exhibited no noticeable differences in marginal adaptation compared to other materials, the observed decline over time may indicate the need for closer evaluation in extended follow-up periods.

After three years of follow-up, SFRC restorations demonstrated favorable functionality in terms of material fracture and retention throughout the observation period. This aligns with previous clinical research reporting that SFRC restorations may exhibit acceptable performance regarding gross fractures and anatomic contour [16, 45, 48]. It also comes in coincidence with previous in vitro studies that documented the superior fracture resistance of SFRC [53–57]. This may be attributed to the prevalence of short fibers acting as crack inhibitors, therefore redirecting the crack and redistributing stress from the polymer matrix to the more resilient fibers [58, 59]. Additionally, PMMA's linear chains inside the cross-linked matrix plasticize the matrix, increasing the material's resistance to fracture [59]. Therefore, the random fiber orientation and the semi-interpenetrating network structure of the polymer matrix had a vital role in enhancing the mechanical characteristics of the material [60]. While SFRC did not demonstrate superiority over the other assessed restorative materials, its characteristics suggest that it may be a favorable choice in deep cavities, high-stress bearing areas or teeth with substantial dentin loss, where enhanced fracture resistance and stress absorption are clinically beneficial.

Indirect lab composite restorations also exhibited favorable performance in terms of material fracture and retention, showing comparable clinical outcomes to direct restorations. These outcomes coincides with the results of two previous systematic reviews, concluding similar clinical performance between direct and indirect composite restorations [8, 28]. Furthermore, a previous clinical trial also concluded that indirect lab composite restorations and SFRC restorations performed similarly after one-year follow-up [16]. Conversely, a prior clinical investigation found that indirect composite restorations performed significantly better than direct composite restorations [7]. These findings can be attributed to the secondary polymerization of the indirect lab composite inlays at a high temperature, which enhances the mechanical properties of the material. This process enables initial polymerization shrinkage and release of post-cure stresses prior to intraoral placement of the restoration [7, 40]. The exclusion of bruxism patients may also contribute to these outcomes, as bruxism is a critical risk factor affecting the failure rate of posterior resin composite restorations, leading to restoration fractures [42]. Clinically, indirect lab composites may be preferred in cases requiring precise occlusal morphology and improved esthetic outcomes, particularly when isolation is challenging or patient expectations for surface quality are high.Indirect lab composite restorations also exhibited favorable performance in terms of material fracture and retention, showing comparable clinical outcomes to direct restorations. These outcomes coincides with the results of two previous systematic reviews, concluding similar clinical performance between direct and indirect composite restorations [8, 28]. Furthermore, a previous clinical trial also concluded that indirect lab composite restorations and SFRC restorations performed similarly after one-year follow-up [16]. Conversely, a prior clinical investigation found that indirect composite restorations performed significantly better than direct composite restorations [7]. These findings can be attributed to the secondary polymerization of the indirect lab composite inlays at a high temperature, which enhances the mechanical properties of the material. This process enables initial polymerization shrinkage and release of post-cure stresses prior to intraoral placement of the restoration [7, 40]. The exclusion of bruxism patients may also contribute to these outcomes, as bruxism is a critical risk factor affecting the failure rate of posterior resin composite restorations, leading to restoration fractures [42]. Clinically, indirect lab composites may be preferred in cases requiring precise occlusal morphology and improved esthetic outcomes, particularly when isolation is challenging or patient expectations for surface quality are high.

The results also revealed that microhybrid resin composite restorations exhibited slightly higher incidence of score 2 (7.1%) according to the utilized FDI criteria, without clinically notable differences when considered alongside the other materials. Additionally, at the three-year evaluation, one microhybrid resin composite restoration demonstrated chipping fractures that were deemed clinically unsatisfactory (score 4), demanding repair. This correlates with the results of a previous clinical study, reporting the complete fracture of a microhybrid resin composite restoration after one year, scoring Charlie, according to USPHS criteria [48]. Furthermore, a prior clinical research concluded that 4.8% of microhybrid resin composite restorations showed minor anatomic form changes after three years of evaluation [34]. These findings also align with the results of previous laboratory investigations which reported that conventional resin composite revealed inferior fracture resistance when compared with SFRC restorations [21, 57, 60]. The observed outcomes probably result from polymerization shrinkage-induced stresses, which accumulate within the restored tooth [6]. Additionally, unfavorable stress transfer between the resin matrix and filler particles, along with the incorporation of pre-polymerized filler particles in conventional hybrid resin composites, could result in reduced mechanical properties [10]. Despite these limitations, microhybrid resin composites remain a viable choice for uncomplicated restorations in low caries-risk patients, particularly when a single-visit, cost-effective solution with simplified technique is clinically appropriate. The clinically acceptable proximal contact and contour observed in the current investigation across all follow-up periods can be attributed to the effective utilization of sectional matrix systems, which have demonstrated an ability to enhance the anatomical accuracy of proximal contacts while preventing excessive tightness [61].

Regarding postoperative sensitivity, it was demonstrated that indirect lab composite restorations exhibited no sensitivity across all assessment periods; nevertheless, two SFRC and three microhybrid resin composite restorations revealed transient postoperative sensitivity that resolved over time as reported by patients. According to the literature, clinical cavity depth has been identified as the primary factor significantly influencing the occurrence of postoperative sensitivity, with sensitivity levels increasing in relation to the depth of the cavities [62]. The observed findings can be attributed to that all cavity depths of indirect lab composite in the current study were moderate, in contrast to the other restorations which exhibited postoperative sensitivity and involved deep cavities. This aligns with the findings from a previous study, which reported mild postoperative sensitivity associated with one SFRC and two indirect lab composite restorations, with similar clinical outcomes reported for both groups [16]. However, another study documented that postoperative sensitivity necessitated endodontic intervention for one SFRC restoration after an 18-month follow-up period, probably related to the patient's low pain perception, or cavity depth compromising the pulp [47].

Concerning secondary caries, the results demonstrated the absence of carious lesions around the margins of the assessed restorations. This is in accordance with previous clinical studies that have similarly reported the absence of secondary caries [16, 34, 45, 47]. The primary determinants impacting the development of secondary caries include patient oral hygiene habits and caries risk [63]. In the current study, participants were advised to maintain optimal oral hygiene and adhere to caries control guidelines, while those identified as high caries risk were excluded from the research. Despite these precautions, individual compliance with oral hygiene instructions and dietary behaviors could not be fully controlled. Moreover, additional confounding variables such as parafunctional habits, occlusal forces, and dietary patterns were not formally assessed but may also influence clinical outcomes and should be considered in upcoming research to better understand their impact on restoration longevity. The aforementioned findings may also be influenced by the delayed development and consequent late-onset of secondary caries, as it is typically detected after five years of restorative treatment [64]. In contrast to the present findings, a previous research [38] documented one SFRC restoration failure due to secondary caries, while another study [58] reported both a repair and a replacement of two SFRC restorations for the same reason, possibly affected by patient-related factors such as improper oral hygiene during the assessment period.

The surface quality of resin composite restorations is intimately correlated to the finishing and polishing procedures, as well as the intrinsic material properties, including the organic matrix and inorganic filler [65, 66]. According to surface luster and texture results, all evaluated restorations revealed acceptable clinical performance with comparable outcomes observed across assessment periods. These favorable outcomes could be ascribed to the finishing and polishing protocols implemented for all restorations that guaranteed a durable smooth surface [16]. Indirect lab composite restorations achieved optimal surface texture and luster (100% scoring 1) during a three-year assessment, in accordance with a prior research [16]. This outcome may be ascribed to their high filler content, which enhances wear resistance and smoothness, and their controlled polymerization process, which reduces shrinkage and air bubble formation that could compromise surface quality [67]. Nevertheless, two SFRC restorations (7.1%) and three microhybrid resin composite restorations (10.7%) revealed clinically acceptable minimal surface alterations, consistent with the findings of two previous studies [34, 47]. The surface roughness of SFRC restorations depends mainly on the microhybrid resin composite used for its veneering, which in both instances its organic content included urethane dimethacrylate (UDMA) rather than bisphenol A-glycidyl methacrylate (Bis-GMA).[58] UDMA-based resin composites are softer than Bis-GMA composites, due to variations in molecular rigidity, ultimate strength, and polymerization rate, which may have contributed to the surface texture changes [34, 58].

Marginal discoloration results from irregularities at the tooth-restoration interface, which accelerates the accumulation of food, beverage stains, and bacterial biofilm in the defects between cavity margins and the restorations [68]. It is also influenced by the material's physical and mechanical properties, such as polymerization shrinkage, modulus of elasticity, and the coefficient of thermal expansion [66]. The results of marginal staining assessment in the current study indicated notable differences across the evaluation periods for the three restorative materials. This aligns with the findings of previous research, reporting that SFRC and conventional resin composite restorations exhibited marginal discoloration that significantly increased over time [34, 47]. These findings may be attributed to the high viscosity of the microhybrid resin composite layer, used both independently and as a veneering layer for SFRC restorations, which could have resulted in defective adaptation, leading to initial marginal leakage at the tooth-restoration interface and subsequent marginal discoloration [48, 58]. Also, inadequate bonding of indirect lab composite can result in marginal gaps and staining [67, 69]. The masticatory stresses and temperature fluctuations in the oral cavity, as well as the patients' diet and oral hygiene habits, may have accelerated this process [19].

After three years, minimal color alterations were noted in 7.1% of SFRC restorations and 10.7% of microhybrid resin composite restorations; both remained clinically acceptable, whereas indirect lab composite restorations revealed no color changes. These outcomes align with a previous study, reporting that only two SFRC restorations exhibited minor color changes at the end of 2 years follow-up [47]. Furthermore, a three-year clinical trial demonstrated that color alterations for both SFRC and conventional resin composite restorations were clinically acceptable and amounted to less than 10% [34]. The staining susceptibility of resin composites can be assigned to their degree of water sorption and the hydrophilicity of the matrix resin, allowing them to absorb not only water but also other fluids, leading to discoloration [70]. The aforementioned findings may be attributable to the microhybrid resin composite used, both independently and for covering SFRC restorations, which is based on UDMA and Bis-GMA free, exhibiting lower water sorption than Bis-GMA, thereby contributing to enhanced stain resistance [34]. Despite the use of the same veneering material in both SFRC and microhybrid resin composite restorations, the slightly higher percentage of ideal color match in the SFRC group may be explained by differences in clinical parameters such as cavity depth and configuration, which can influence light transmission and final shade perception [71]. Additionally, patient-related factors such as diet, oral hygiene practices, and exposure to staining agents over time may have variably affected long-term color stability across groups, contributing to the observed differences. These results disagreed with previous studies reporting the inferior color stability of SFRC restorations compared to both indirect restorations and conventional resin composites [16, 48] This contradiction may be attributed to the presence of residual amalgam tattoos or dark sclerotic dentin at the base of the cavities in discolored restorations, which could not be concealed due to the high translucency of the SFRC material [53].

The limitations of this study included the inability to implement operator blinding owing to the distinct restorative procedures utilized, which may have introduced potential bias. The relatively narrow age range of participants (18–35 years) may limit the generalizability of the findings to older populations who may present different oral conditions and restorative demands. A further concern is the absence of an additional buffer for potential dropouts, which, together with the limited sample size and observed attrition, could have affected the statistical power and robustness of the findings if further losses had occurred. The split-mouth design occasionally required placing two restorations with different materials in the same quadrant, which may have limited patients’ ability to precisely localize discomfort and introduced potential symptom misattribution. Another methodological limitation is the clinical judgment applied in the documented case example, where multiple untreated carious lesions were present in non-study teeth, including tooth #26, which exhibited a deep carious lesion as revealed by the postoperative bitewing radiograph (Fig. 1). At recruitment, this patient was classified as having moderate caries risk, and tooth #26 was asymptomatic, based only on clinical examination in accordance with the study protocol. However, the absence of baseline radiographic screening might have contributed to underestimating the actual caries risk in this case, as some lesions were not clinically detectable. The untreated lesions were scheduled for appropriate management following their identification; nonetheless, we acknowledge that, in the context of a clinical trial, the presence of untreated lesions could introduce a potential risk of unforeseeable symptoms during follow-up, which might confound patient-reported outcomes. Future clinical trials should incorporate broader age representation and additional buffers for potential dropouts to enhance the assessment of long-term material performance. Baseline radiographs should also be included to ensure accurate caries risk evaluation, particularly in contexts where misclassification could affect data interpretation. Study designs should further minimize risks associated with untreated carious lesions in non-study teeth to prevent unexpected complaints and ensure reliable evaluation of postoperative pain in study teeth.

## Conclusion

After a three-year follow-up period, both SFRC and indirect lab composite demonstrated acceptable clinical performance, comparable to that of microhybrid resin composite, as evaluated by the FDI criteria.

## Supplementary Information

Below is the link to the electronic supplementary material.

Supplementary file 1 (PDF 76.0 KB

## Data Availability

The datasets generated and analyzed in the current study, as well as the full trial protocol, are available from corresponding author upon reasonable request.
