# Supplementary material for: Clinical performance of short fiber-reinforced and indirect resin composites in class I and class II restorations: A three-year randomized clinical trial
Source: Clin Oral Investig. 2025 Nov 21;29(12):580. doi: 10.1007/s00784-025-06593-x (PMC12638419; doi:10.1007/s00784-025-06593-x)
Supplement: Supplementary file 1 — (PDF 76.0 KB [file 784_2025_6593_MOESM1_ESM.pdf]

ClinicalTrials.gov PRS
Protocol Registration and Results System

ClinicalTrials.gov Protocol Registration and Results System (PRS) Receipt
Release Date: January 27, 2025

ClinicalTrials.gov ID: NCT06803537

Study Identification

Unique Protocol ID: A02071221

Brief Title: Three-Year Clinical Performance of Fiber-reinforced Versus Indirect Resin Composite Posterior Restorations

Official Title: Three-Year Clinical Performance of Fiber-reinforced Versus Indirect Resin Composite Posterior Restorations

Secondary IDs:

Study Status

Record Verification: January 2025

Overall Status: Active, not recruiting

Study Start: January 1, 2022 [Actual]

Primary Completion: February 2025 [Anticipated]

Study Completion: April 2025 [Anticipated]

Sponsor/Collaborators

Sponsor: Mansoura University

Responsible Party: Sponsor

Collaborators:

Oversight

U.S. FDA-regulated Drug: No

U.S. FDA-regulated Device: No

U.S. FDA IND/IDE: No

Human Subjects Review: Board Status: Approved
Approval Number: A02071221
Board Name: The ethics committee of Mansoura University
Board Affiliation: The ethics committee of Mansoura University
Phone: 01000235774
Email: denrasha91@gmail.com
Address:

mansoura

Data Monitoring: No

Study Description

Brief Summary: This study aimed to perform a comparison between the clinical performance of short fiber-reinforced composite and indirect lab composite restorations in posterior dentition.

Detailed Description:

Conditions

Conditions: Occlusal Caries
Proximal Caries

Keywords: fiber-reinforced composite
indirect lab composite
posterior restorations

Study Design

Study Type: Interventional

Primary Purpose: Treatment

Study Phase: N/A

Interventional Study Model: Parallel Assignment

Number of Arms: 3

Masking: Double (Participant, Outcomes Assessor)

Allocation: Randomized

Enrollment: 33 [Actual]

Arms and Interventions

Arms	Assigned Interventions
Active Comparator: Fiber-reinforced composite	indirect lab composite<br>indirect lab processed resin composite inlay restorations<br>Conventional resin composite<br>conventional microhybrid resin composite posterior restorations
Active Comparator: Indirect lab composite	Fiber-reinforced composite<br>short fiber-reinforced composite used as base covered by conventional composite<br>Conventional resin composite<br>conventional microhybrid resin composite posterior restorations
Active Comparator: Conventional microhybrid resin composite	Fiber-reinforced composite<br>short fiber-reinforced composite used as base covered by conventional composite<br>indirect lab composite<br>indirect lab processed resin composite inlay restorations

Outcome Measures

Primary Outcome Measure:

1. FDI criteria for evaluation
   [Time Frame: Three years follow-up]

Eligibility

Minimum Age: 20 Years

Maximum Age: 35 Years

Sex: All

Gender Based:

Accepts Healthy Volunteers: No

Criteria: Inclusion Criteria:

1. Patients aged 20–35 year from both genders.
2. Good oral hygiene: patients with low and moderate caries risk (according to Caries management by caries risk assessment CAMBRA sheets) were enrolled in this study.
3. The visual examination should reveal the presence of a minimum of three primary occlusal or proximal caries (Black Class I and II) with a severity score of 4 or 5 according to the International Caries Detection and Assessment System (ICDAS).
4. The carious teeth must exhibit vitality and do not display periapical radiolucency as evaluated by periapical radiography.
5. The selected teeth must be under stable occlusion.

Exclusion Criteria:

1. Uncontrolled systemic disease
2. Extremely poor oral hygiene
3. Chronic periodontitis
4. Heavy bruxism
5. Cavities with a buccolingual width exceeding two-thirds of the intercuspal distance or requiring cusp covering.
6. Patients undergoing orthodontic procedures.
7. Patients unable to attend the scheduled recall appointments.

Contacts/Locations

Central Contact Person: Rasha Salama
Telephone: 01000235774
Email: denrasha91@gmail.com

Central Contact Backup:

Study Officials:

Locations: **Egypt**
Mansoura university
mansoura, Dakahlia, Egypt, 35516
Contact: Rasha salama 01000235774 denrasha91@gmail.com

IPDSharing

Plan to Share IPD:

References

Citations:

Links:

Available IPD/Information:
